# Amine‐Acrylate Liquid Single Crystal Elastomers Reinforced by Hydrogen Bonding

**DOI:** 10.1002/adma.202101955

**Published:** 2021-06-18

**Authors:** Weike Zou, Xueyan Lin, Eugene M. Terentjev

**Affiliations:** ^1^ State Key Laboratory of Chemical Engineering College of Chemical and Biological Engineering Zhejiang University Hangzhou 310027 P. R. China; ^2^ Cavendish Laboratory University of Cambridge J. J. Thomson Avenue Cambridge CB3 0HE UK

**Keywords:** liquid crystalline elastomers, aza‐Michael addition, hydrogen bonding

## Abstract

Liquid crystalline elastomers (LCEs) have been considered one of the most promising material concepts for artificial muscles. However, accomplishing actuation of LCEs requires macroscopic alignment of the liquid‐crystalline orientation in the rubbery network, which imposes challenges in the materials chemistry and processing. A two‐stage curing strategy has been the dominating approach during last three decades. Despite its many successes, the method is difficult in practice and requires delicate experiential skills, dealing with intrinsic fragility of intermediate gels after the first crosslinking stage. Here, a robust fabrication method for monodomain LCE based on the amine‐acrylate aza‐Michael addition is developed, involving two readily commercially available components with no catalyst. The method is based on the large kinetic difference of hydrogen addition in primary amines to acrylates, which offers a sufficient gap separating two stages of curing and enabling versatile mechanical alignment techniques for manufacturing monodomain LCE in both liquid and gel states. Importantly, the mechanically robust network, helping processability at a partial‐crosslinking stage, is facilitated by the chemically generated hydrogen bonding all through the process, as a by‐product of hydrogen addition. Such a facile two‐component kit‐like fabrication should aid researchers from various fields in the search for a practical and reliable process of making soft actuators.

## Graphical abstract

A new chemical approach to fabricating monodomain LCE based on aza‐Michael addition is developed, compatible with both liquid and gel alignment techniques, to manufacture actuators with versatile shapes. The facile process involving simply mixing two components is enabled by the two‐stage differentiated kinetics of primary amines toward acrylates, generating hydrogen bonding, enhancing the mechanical properties of the elastomer.

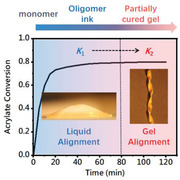

## 1 Introduction

Liquid crystalline elastomers (LCEs) are intelligent soft materials, achieving remarkable shape‐morphing,^[^ ^1^ ^]^ exploiting interplay between the LC phase change and the polymeric entropic elasticity.^[^ ^2^, ^3^, ^4^, ^5^, ^6^, ^7^ ^]^ In a technological sense, preparation of monodomain (permanently aligned “single‐crystal”) LCE is usually required as the key protocol to obtain muscle‐like actuation with artificial soft materials.^[^ ^1^, ^2^, ^3^ ^]^ The need for such physically biased molecular configuration, however, imposes technical challenges for a classical synthesis of polymeric elastomers due to their isotropic nature. In 1991, Finkelmann et al.^[^ ^8^ ^]^ introduced a two‐stage hydrosilylation approach and reported the first successful “nematic liquid single crystal elastomers” with free‐standing actuation. In this approach, the essence of which has been the preferred protocol for fabrication of monodomain LCE for the next two decades, a uniaxial mechanical extension was applied to a lightly crosslinked gel, to establish the internal uniaxial alignment field, which was followed by further (second‐stage) curing to permanently fix that alignment. Yet this protocol is very difficult in practice, because of the intrinsic mechanical fragility of half‐cured gels that need to be stretched sufficiently to impose the alignment. This has diminished the accessibility of LCEs to be readily utilized in ever‐expanding applications involving shape‐morphing and actuation.

Aimed at more complex patterns of LC alignment and circumventing the staged curing problems, other techniques based on external fields have been developed, notably the surface alignment^[^ ^9^, ^10^, ^11^, ^12^ ^]^ and dynamic bond exchange.^[^ ^13^, ^14^, ^15^, ^16^, ^17^, ^18^, ^19^, ^20^ ^]^ The diverse pixel‐defined surface of the substrate enabled a great expansion of actuation modes other than simple contraction‐extension. Despite the functionalization, the scale of the materials was nevertheless confined by specified substrates, and the limited depth of surface penetration into the bulk of the LCE rendered the method technological insufficient for fabricating at large scales. Therefore, for a general and flexible LCE fabrication, mechanical stretching remains the simplest strategy to produce monodomain LCE in versatile functional forms. For instance, this is prominent in woven fibers given the well‐established methods of polymer fiber processing. New chemistry, other than hydrosilylation, was desired that allowed for robust reactions and convenient ways of mechanical alignment. The commercial availability of diacrylate reactive mesogens, such as RM257 and RM82, has been a strong driver in the LCE field in recent years, offering a satisfying alternative considering a library of benign reactions involving diacrylates. Particularly, a thiol‐acrylate addition approach, first developed by Yakacki et al. in 2015,^[^ ^21^ ^]^ quickly gained its popularity due to its overwhelming ease over the classical Finkelmann method. The orthogonal reactions of thiol‐acrylate and acrylate self‐polymerization, enabled by thermal and UV radiation, respectively, constituted the two stages for curing LCE, with much more stable mechanical stretching conditions. The mandatory introduction of irradiation brought additional challenges, such as penetration depth and homogeneity, both imposing limits on the dimensions available and limiting the fabrication. Recently Zhang et al, aiming for flexible welding, have deployed thermal second‐stage thermal crosslinking as an alternative.^[^ ^22^ ^]^ Yet the necessary preloading of initiators, both UV and thermal, required specific care for deterring undesired initiator decomposition during fabrication or storage.

**Figure 1:**
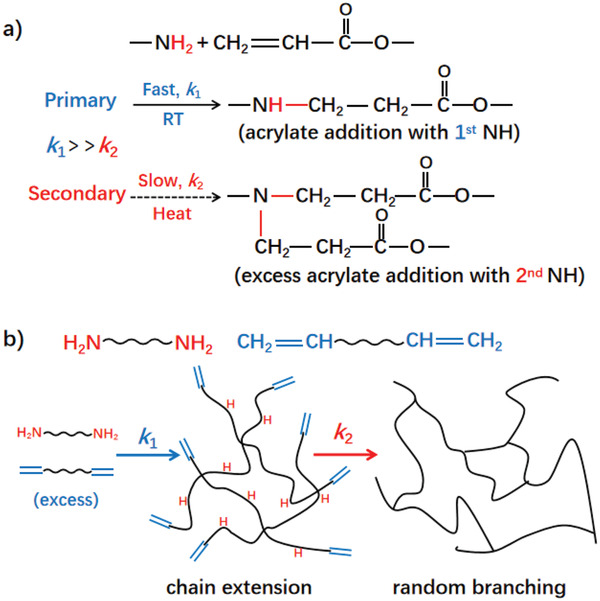
Mechanism of amine‐acrylate aza‐Michael addition for curing polymer networks. a) Different addition kinetics of 1st and 2nd hydrogen of aliphatic primary amines towards acrylates. b) Differentiated kinetics resulted in sequential stages for diacrylates reacting with diamines. While the 1st hydrogen basically participated in chain extension, the remaining 2nd hydrogen would initiate random branching with excessive acrylates to cure the network.

Nevertheless, important insights could be deduced from this history. In the Finkelmann approach, the genuine breakthrough was the introduction of an asymmetrical crosslinker, ending respectively with vinyl and methacrylate groups, which exhibited remarkably different reactivity towards hydrogen addition.^[^ ^8^ ^]^ Thus during the crosslinking, the large kinetic difference, in fact, provided the natural cut‐off in the curing process between two stages featuring distinct reactions: the first fast stage, generating oligomers or a lightly cured gel, and then the slow stage, providing a sufficient processing window for mechanical treatment. Therefore, to match the physical manifestation of induced macroscopic anisotropy, a similar chemical orthogonality is required, either via distinct triggers, or via sufficient kinetic difference if only heat is available. We found that amine‐acrylate Michael addition happens to perfectly fit for this very scenario. While sharing the efficient nature of “click” Michael addition with thiol‐acrylate, the self‐catalyzing amine‐acrylate allows for a more concise ingredient recipe. Yet the most distinguishing feature is that the amine‐acrylate addition with primary amines is intrinsically a two‐stage reaction with a large kinetic difference between the primary hydrogen (fast, *k*_1_) and the generated secondary hydrogen (slow, *k*_2_) reacting with acrylates (**Figure** **1**a).^[^ ^22^, ^23^, ^24^ ^]^ As Retailleau et al. reported in the kinetic study of diacrylates with diamines,^[^ ^22^ ^]^ the first hydrogen was almost completely consumed before the second hydrogen slowly began to participate in the addition, indicating a *k*_1_ about two orders magnitude greater than *k*_2_.^[^ ^25^ ^]^ This unique chemical transition thus allowed facile preparation of oligomers which could be autonomously crosslinked as time evolved (Figure 1b). These chemical advantages inspired us to fabricate monodomain LCEs with remarkable ease, by simply mixing primary diamines and diacrylate mesogens, under mild heating, instead of employing a thiol‐acrylate recipe involving additional catalysts, crosslinkers, initiators, and a UV apparatus.^[^ ^26^ ^]^ It should be noted that Ware et al. have utilized the amine‐acrylate reaction in a series of recent publications,^[^ ^27^, ^28^, ^29^ ^]^ however, the monofunctional primary amine was used as chain extender alone. The second‐stage crosslinking was still accomplished via self‐polymerization of excessive acrylates under UV irradiation. Here we emphasize that using diamines with four hierarchically functional hydrogens could play both roles: as chain‐extenders and crosslinkers, in succession. The differences will be self‐explanatory following the illustration below.

## 2 Result and Discussions

**Figure 2:**
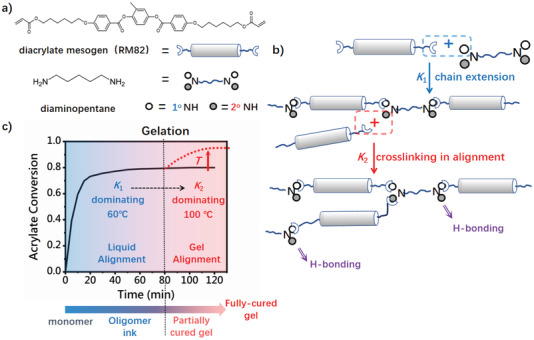
Composition and reactions in amine‐acrylate LCEs. a) Monomers used in the synthesis. b) Preparation sequence of monodomain LCEs. Mechanical alignment was applied to the partially cured gel for further complete curing. c) Conversion of diacrylates with time. The process could be divided into two stages representing chain extension and crosslinking respectively, yielding LC oligomers ink and cured LC gel.

To demonstrate this chemical concept in the simplest possible way, we employed a combination of RM82 and 1,5‐diaminopentane (**Figure** **2**a), although other readily available diacrylate mesogens like RM257, and various aliphatic diamines with different length, should work equally well for more refined property tailoring. Upon mixing, the addition between diamines and diacrylates is dominated by chain extension at the beginning, basically taking diamines as dithiol‐like chain extenders (Figure 2b). Ideally, once the 1° amines are consumed in completion of this first reaction stage, and acrylates are still in excess, crosslinking by branching of generated 2° amine with acrylate‐ended oligomers would then proceed. Importantly, the excess of acrylate functional groups theoretically demanded a molar ratio of diamine/diacrylate, referred as *r*, to be less than 1 for gelation, although in practice we found that *r* ≥ 1 would also lead to gel as the crosslinking was always proceeding in a competitive way with chain extension concerning the specific environment (temperature, solvent). To ensure a moderate crosslinking density compatible with LCE actuation, *r* = 0.9 was kept constant in the following experiments, although one could achieve a much higher density of network crosslinking, and therefore mechanical strength of the material, by reducing this ratio further.

Since high temperature would diminish the kinetic difference between the 1° and 2° hydrogen addition, the first‐stage reaction should ideally be conducted at as low a temperature as possible, to enhance the cut‐off. In order to maintain a homogeneous mixture, the reaction was carried out at 60 °C with a necessary amount of solvent (DMF 25 wt%) depressing the LC phase (Figure 2c). The two‐stage addition feature could be confirmed by analyzing acrylate conversion together with amine consumption obtained from the Fourier transform infrared (FTIR) spectroscopy study as shown in Figures S1 and S2, Supporting Information. A clear reduction in acrylate conversion rate could be observed within 0.5 h, indicating a paradigm transition. As 1° hydrogen conversion was driven to completion yielding oligomers, the 2° hydrogen conversion leading to chain branching became equally significant, although much more slow. Usually a partially cured elastomer could be obtained in 1.5 h, after which the material could be efficiently handled and stored in the dark for future treatment. Such well‐defined liquid–gel transition cut‐off allowed us to exploit the processing windows to fabricate versatile forms of LCE at different scales, utilizing alignment techniques that are usually mutually exclusive (Figure 2c). That is, prior to gelation, the LC oligomers could be treated as “liquid ink” for extrusion 3D printing, which generated patterned alignment from shear strain. On the other hand, after gelation, a classical mechanical‐stretching approach was yielding monodomain LCE, as will be introduced first.

**Figure 3:**
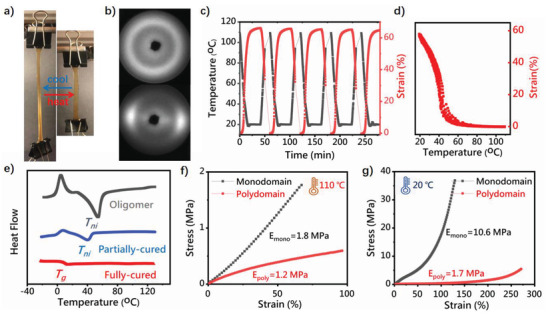
Characterization of LCE. a) Visual illustration of actuation in monodomain LCE. b) X‐ray diffraction pattern of monodomain and polydomain LCE. c) Repeated actuation cycles of monodomain LCE at a rate 10° min^−1^. d) The repeated strain–temperature curves on cooling, from (c). e) DSC scanning of oligomer, partially cured and fully cured LCE samples on heating. f,g) Stress–strain curves of monodomain and polydomain LCE above and below the nematic transition, obtained at a high stretching rate of 50% min^−1^.

Upon gelation, the partially cured gel exhibited quite robust mechanical properties, and could be easily stretched to over 900% at room temperature (Figure S3, Supporting Information). Given the very low initial crosslinking density, such high stretchability was supported by the pervasive hydrogen bonding generated between N—H and carbonyl groups on the oligomer chains as indicated by a large fraction of bonded NH versus free NH in FTIR spectroscopy (Figure S4, Supporting Information), which enabled the robust and flexible process for mechanical alignment. The stretched sample was thereafter heated to 90 °C for 12 h to facilitate the crosslinking completion. The resulting monodomain LC alignment could be easily confirmed by its clear transparent appearance, X‐ray characterization, and the fully reversible actuation upon temperature variation (**Figure** **3**a). The analysis of X‐ray scattering images (Figure 3b) indicated the nematic liquid crystalline order with an order parameter of ca. 0.55 for the sample with 200% strain during the second‐stage curing in the isotropic phase, while the control sample without mechanical loading remained isotropic in the diffraction pattern, and white in appearance as expected for a polydomain LCE (Figure S5, Supporting Information). Multiple actuation cycles show that the aligned LCE sample changes its natural length between 20 and 110 °C with a consistent amplitude of over 60% (Figure 3c). While the temperature–strain curve indicated a nematic‐isotropic transition of about 40 °C (Figure 3d), only a glass transition of 10 °C was detected in differential calorimetry (DSC) scanning in the fully cured LCE. Intriguingly, for the LC oligomer ink and for the partially cured sample, a nematic transition around 40 °C was clearly revealed (Figure 3e). We attribute the disappearance of *T*_ni_ to the effect of hydrogen bonding, which was fully activated between chains as DMF was evaporated. In contrast, in oligomers and partially cured networks, the hydrogen bonding could be screened by the polar DMF solvent, thus allowing sufficient mobility of mesogenic groups to register the entropy change on the nematic transition. The enhancement of mechanical strength by hydrogen bonding could also be observed from tensile tests on monodomain and polydomain samples (Figure 3f,g): the elastomer in the isotropic phase still exhibited an elastic modulus of 1.8 MPa, surpassing most reported values and thus providing a considerable capacity for mechanical work. While in the nematic phase, the alignment was mechanically manifested in a steep stress–strain curve (no soft elasticity as in polydomain sample) reaching a breaking strength of ≈40 MPa, which is comparable to commercial hydrogen‐rich neat thermoplastic polyurethane.^[^ ^30^ ^]^

**Figure 4:**
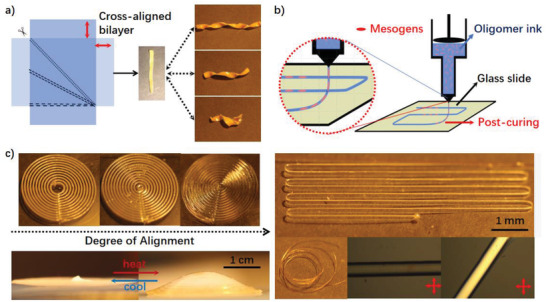
a) Bilayer strategy for fabricating actuators with a versatile twist via welded cross‐aligned LCEs. b) Scheme of printing LC oligomer ink to deposit aligned LCE fibers. c) Manipulation of alignment degree and pattern to achieve complex LCE actuators, the alignment in each filament illustrated by polarized microscopy.

The outlined benefits of amine‐acrylate LCE naturally brought added convenience for their mechanical treatment. Apart from the simple stretching alignment, a bilayer LCE with crossed alignment was produced, utilizing the welding potential during the second‐stage crosslinking. Specifically, two lightly crosslinked LCE gels were stretched to 200% and aligned in crossed fashion on a flat substrate, with clamps fixing the bilayers in close contact. During the second‐stage curing, molecular networks of each layer were interconnected via chemical bonding across the interface, while preserving the respective alignment in the bulk of each film. The assembly of differently aligned LCE bilayers thus provided coiled actuators with pitch programmability via the oriented cut‐through, following the analysis of bilayer shape instabilities,^[^ ^31^ ^]^ expanding the general scope of traditional actuation modes with a range of elastic instability regimes (**Figure** **4**a).

Given that the alignment techniques conducted at smaller scales were nonetheless demanded for action versatility, an increasingly popular 3D printing approach^[^ ^32^, ^33^, ^34^, ^35^, ^36^, ^37^ ^]^ based on shear alignment of LC was also investigated. As previously mentioned, the processing window for oligomers as “liquid ink” could endure for as long as 1 h. When the viscous LC oligomer was extruded out of the syringe equipped with a mechanical piston, molecular rearrangement by the shear flow would yield a reasonably well‐aligned LC filament as indicated by its transparency. While previous reports on 3D printing LCE relied on in situ UV crosslinking to fix the alignment, our extruded LC filament supported on the glass slide could retain the shear‐induced alignment, as DMF evaporation activated the hydrogen bonding as a temporary fixing measure, until further thermal curing is completed as in the gel approach. As indicated by polarized optical microscope (Figure 4c), these uniform filaments fully preserved the alignment achieved on extrusion, and could be used as monodomain LCE fibers when peeled off. Intriguingly, it was also found that decreasing the gap distance between the syringe and substrate (keeping other parameters constant) would impose an additional effect on the alignment of LC filaments. The enhanced transparency clearly illustrated an increase in alignment as the gap distance decreased, so that additional laminar shear was produced by the nozzle tip on the extruded polymer. Below a certain distance, the linear filaments would merge into a continuous thin 2D plane layer, with the embedded 3D shape programmability. For instance, a layout of concentric filaments would eventually transform into a cone with positive Gaussian curvature as shown in Figure 4c. An independent study on adjusting printing parameters to manage the alignment degree is under way.

## 3 Conclusion

We have investigated a new chemical approach towards monodomain “single crystal” LCE fabrication, which is compatible with both classical mechanical stretching and 3D printing techniques. The fabrication required simple mixing of only two commercially available components, followed by mild heating, which should be accessible to a general materials community. The essence of our work lies in the rediscovery of the two‐stage kinetic nature of the amine‐acrylate aza‐Michael addition. Less known compared to its thiol counterpart, this reaction could find its unique application spectrum in monodomain LCE fabrication, a process that has widely been considered delicate and lacking robustness ever since the Finkelmann method was established. From the material perspective, the generated poly(amino‐ester), the condensed product from diamines and diacrylates, exhibits a distinct feature as hydrogen bonding medium, setting amines apart from thiols. Hydrogen bonding has been a well‐recognized toughening strategy in polymeric materials providing an alternative approach for energy dissipation through chain interaction enhancement. As a trade‐off, the incorporation of these entities along the polymer chains would inevitably cost the rubber flexibility, as in the cases of polyurea, polyamide, or polyurethane where a well‐defined phase separation is required for preserving high extensibility of soft domains. Intriguingly, the β‐amino esters obtained from aza‐Michael addition provide a much milder hydrogen bonding intensity, as the receptors and donors are separated by two carbons instead of being rigidly well‐packaged. Such a toughening strategy would nonetheless be beneficial for the mechanical performance of LCE, allowing for a high output of work on actuation as well as tensile strength as observed. Therefore it is reasonable to believe that the potential of amines as alternatives for thiols could bring new opportunities for the smart‐materials community.

## 4 Experimental Section

### Materials and Preparation of LCE

1,4‐Bis‐[4‐(6‐acryloyloxyhexyloxy)benzoyloxy]‐2‐methylbenzene (RM82) was purchased from Daken Chemical Ltd. 1,5‐diaminopentane was purchased from TCI. DMF was purchased from Sigma‐Aldrich. Typically, 1.68 g of RM82 was dissolved in 0.5 g of DMF and was melted at 80 °C to be completely dissolved. Then 0.23 g of 1,5‐diaminopentane was added and mixed to obtain a homogeneous precursor solution. The solution was then poured into a glass dish for curing at 60 °C with a lid. For liquid printing, DMF solution could be reduced to 0.25 g for better alignment and taken as printable ink after 0.5 h. For gel alignment, the LC gel was formed after 1.5 h and could be taken out of the dish and stretched to 200% for further curing at 90 °C for another 12 h.

We found that the ester groups in the middle of RM82 mesogen had a tendency to degrade at high temperature in the presence of un‐reacted amines, and break the rod‐like mesogen into fragments of terminal —OH and amide, which they could see by increasing the yellow color (and of course by deterioration of nematic LC phase). On the other hand, while in the nematic phase (below 90 °C), the ester groups were protected by the packing of rods. This was why we could not carry out the second‐stage crosslinking at a temperature higher than 90 °C or keep the sample for a long time even at that temperature. As a result, their monodomain LCE was nematic‐genesis.

### Characterization

DSC4000 from PerkinElmer was used to obtain the transition temperatures all with a heating rate of 10 °C min^−1^. DMA Discovery 850 from TA instruments was used to characterize the mechanical properties with rectangular samples mounted on the clamp. A Philips Xray generator PW1830 with the wavelength of 0.154 nm, equipped by a wide‐area CCD detector from Photonic Sciences Ltd was used for characterizing alignment of LCE. The distance between the sample and the imaging area was 100 mm. FTIR spectroscopy was used to monitor the acrylate conversion during times and the peaks during acrylate (around 810 cm^–1^) were integrated to obtain the conversion data.

Printing LCE filaments was conducted on a customized DIW 3D printer (Hyrel KR‐15 engine) with computer controlled syringe extrusion. The initial mixture of RM82 and diamine dissolved in DMF was transferred to into the syringe (KR2, Hyrel3D) and allowed to react for 1 h before extrusion. The oligomer was then extruded through the nozzle (400 µm diameter) at a constant flow rate of about 2 (rectangular filament) and 4 µl min^−1^ (circular filament) onto the substrate. As the flow rate was effectively a product of the path width, path height, and the printing speed, adjusting the height of printing would change the width of the final filament. For the circle as in Figure 4c, the flow rate was kept constant while the path height was decreased, as the distance between the substrate and the nozzle was shortened from 0.25 to 0.2, to 0.15 mm from left to right as in Figure 4c. As a result, the circular filament became wider and finally fused with its parallel neighbors to make a continuous flat film.

## Conflict of Interest

The authors declare no conflict of interest.

## Supporting information

Supporting Information

## Data Availability

The data that support the findings of this study are available from the corresponding author upon reasonable request.

## References

[1] M. Warner , E. M. Terentjev , Liquid Crystal Elastomers, Oxford University Press, Oxford, UK 2007.

[2] T. J. White , D. J. Broer , Nat. Mater. 2015, 14, 1087. 26490216 10.1038/nmat4433

[3] C. Ohm , M. Brehmer , R. Zentel , Adv. Mater. 2010, 22, 3366. 20512812 10.1002/adma.200904059

[4] G. Vantomme , L. C. Elands , A. H. Gelebart , E. Meijer , A. Y. Pogromsky , H. Nijmeijer , D. J. Broer , Nat. Mater. 2021, 10.1038/s41563-021-00931-6. PMC7612044 33603183

[5] J. Lv , Y. Liu , J. Wei , E. Chen , L. Qin , Y. Yu , Nature 2016, 537, 179. 27604946 10.1038/nature19344

[6] A. S. Künstler , H. Kim , R. C. Hayward , Adv. Mater. 2019, 31, 1901216. 10.1002/adma.201901216 31012181

[7] H.‐F. Lu , M. Wang , X.‐M. Chen , B.‐P. Lin , H. Yang , J. Am. Chem. Soc. 2019, 141, 14364. 31429282 10.1021/jacs.9b06757

[8] J. Küpfer , H. Finkelmann , Die Makromol. Chem. Rapid Comm. 1991, 12, 717.

[9] L. T. de Haan , C. Sánchez‐Somolinos , C. M. Bastiaansen , A. P. Schenning , D. J. Broer , Angew. Chem., Int. Ed. 2012, 124, 12637. 10.1002/anie.201205964 23124726

[10] T. H. Ware , M. E. McConney , J. J. Wie , V. P. Tondiglia , T. J. White , Science 2015, 347, 982. 25722408 10.1126/science.1261019

[11] Y. Xia , G. Cedillo‐Servin , R. D. Kamien , S. Yang , Adv. Mater. 2016, 28, 9637. 27717070 10.1002/adma.201603751

[12] Y. Xia , X. Zhang , S. Yang , Angew. Chem., Int. Ed. 2018, 130, 5767.

[13] Z. Pei , Y. Yang , Q. Chen , E. M. Terentjev , Y. Wei , Y. Ji , Nat. Mater. 2014, 13, 36. 24292422 10.1038/nmat3812

[14] E. C. Davidson , A. Kotikian , S. Li , J. Aizenberg , J. A. Lewis , Adv. Mater. 2020, 32, 1905682. 10.1002/adma.201905682 31664754

[15] Z. C. Jiang , Y. Y. Xiao , L. Yin , L. Han , Y. Zhao , Angew. Chem., Int. Ed. 2020, 132, 4955.

[16] Z. Wang , H. Tian , Q. He , S. Cai , ACS Appl. Mater. Interfaces 2017, 9, 33119. 28879760 10.1021/acsami.7b09246

[17] M. K. McBride , A. M. Martinez , L. Cox , M. Alim , K. Childress , M. Beiswinger , M. Podgorski , B. T. Worrell , J. Killgore , C. N. Bowman , Sci. Adv. 2018, 4, eaat4634. 30151428 10.1126/sciadv.aat4634 PMC6108565

[18] M. O. Saed , A. Gablier , E. M. Terentejv , Adv. Funct. Mater. 2020, 30, 1906458.

[19] Y. Li , Y. Zhang , O. Rios , J. K. Keum , M. R. Kessler , RSC Adv. 2017, 7, 37248.

[20] M. O. Saed , A. Gablier , E. M. Terentjev , Chem. Rev. 2021, 10.1021/acs.chemrev.0c01057. PMC8915166 33596647

[21] C. M. Yakacki , M. Saed , D. P. Nair , T. Gong , S. M. Reed , C. N. Bowman , RSC Adv. 2015, 5, 18997.

[22] Y. Zhang , Z. Wang , Y. Yang , Q. Chen , X. Qian , Y. Wu , H. Liang , Y. Xu , Y. Wei , Y. Ji , Sci. Adv. 2020, 6, eaay8606. 32158947 10.1126/sciadv.aay8606 PMC7048416

[23] M. Retailleau , A. Ibrahim , C. Croutxé‐Barghorn , X. Allonas , C. Ley , D. Le Nouen , ACS Macro Lett. 2015, 4, 1327. 35614777 10.1021/acsmacrolett.5b00675

[24] G. González , X. Fernández‐Francos , À. Serra , M. Sangermano , X. Ramis , Polym. Chem. 2015, 6, 6987.

[25] A. Genest , D. Portinha , E. Fleury , F. Ganachaud , Prog. Polym. Sci. 2017, 72, 61.

[26] S. k. Ahn , T. H. Ware , K. M. Lee , V. P. Tondiglia , T. J. White , Adv. Funct. Mater. 2016, 26, 5819.

[27] H.‐H. Yoon , D.‐Y. Kim , K.‐U. Jeong , S.‐k. Ahn , Macromolecules 2018, 51, 1141.

[28] R. S. Kularatne , H. Kim , J. M. Boothby , T. H. Ware , J. Polym. Sci., Part B: Polym. Phys. 2017, 55, 395.

[29] C. P. Ambulo , J. J. Burroughs , J. M. Boothby , H. Kim , M. R. Shankar , T. H. Ware , ACS Appl. Mater. Interfaces 2017, 9, 37332. 28967260 10.1021/acsami.7b11851

[30] T. Ware , T. White , Polym. Chem. 2015, 6, 4835.

[31] S. Armon , E. Efrati , R. Kupferman , E. Sharon , Science 2011, 333, 1726. 21940888 10.1126/science.1203874

[32] K. Yao , M. Song , D. Hourston , D. Luo , Polymer 2002, 43, 1017.

[33] A. Kotikian , R. L. Truby , J. W. Boley , T. J. White , J. A. Lewis , Adv. Mater. 2018, 30, 1706164. 10.1002/adma.201706164 29334165

[34] M. O. Saed , C. P. Ambulo , H. Kim , R. De , V. Raval , K. Searles , D. A. Siddiqui , J. M. O. Cue , M. C. Stefan , M. R. Shankar , Adv. Funct. Mater. 2019, 29, 1806412.

[35] M. López‐Valdeolivas , D. Liu , D. J. Broer , C. Sánchez‐Somolinos , Macromol. Rapid Commun. 2018, 39, 1700710. 10.1002/marc.201700710 29210486

[36] J. Liu , Y. Gao , H. Wang , R. Poling‐Skutvik , C. O. Osuji , S. Yang , Adv. Intell. Syst. 2020, 2, 1900163.

[37] D. J. Roach , C. Yuan , X. Kuang , V. C.‐F. Li , P. Blake , M. L. Romero , I. Hammel , K. Yu , H. J. Qi , ACS Appl. Mater. Interfaces 2019, 11, 19514. 31062572 10.1021/acsami.9b04401

