# Supplementary material for: Amine‐Acrylate Liquid Single Crystal Elastomers Reinforced by Hydrogen Bonding
Source: Adv Mater. 2021 Jun 18;33(30):2101955. doi: 10.1002/adma.202101955 (PMC11469043; doi:10.1002/adma.202101955)
Supplement: Supplementary file 1 — Supporting Information [file ADMA-33-2101955-s001.pdf]

© 2021 Wiley-VCH GmbH

ADVANCED MATERIALS

Supporting Information

for *Adv. Mater.,* DOI: 10.1002/adma.202101955

Amine-Acrylate Liquid Single Crystal Elastomers Reinforced by Hydrogen Bonding

Weike Zou, Xueyan Lin, and Eugene M. Terentjev**

Supporting Information

Amine-Acrylate Liquid Single Crystal Elastomers Reinforced by Hydrogen Bonding

Weike Zou, Xueyan Lin and Eugene M. Terenjtev

Figure S1. FTIR spectra monitoring the acrylate conversion during the reaction.

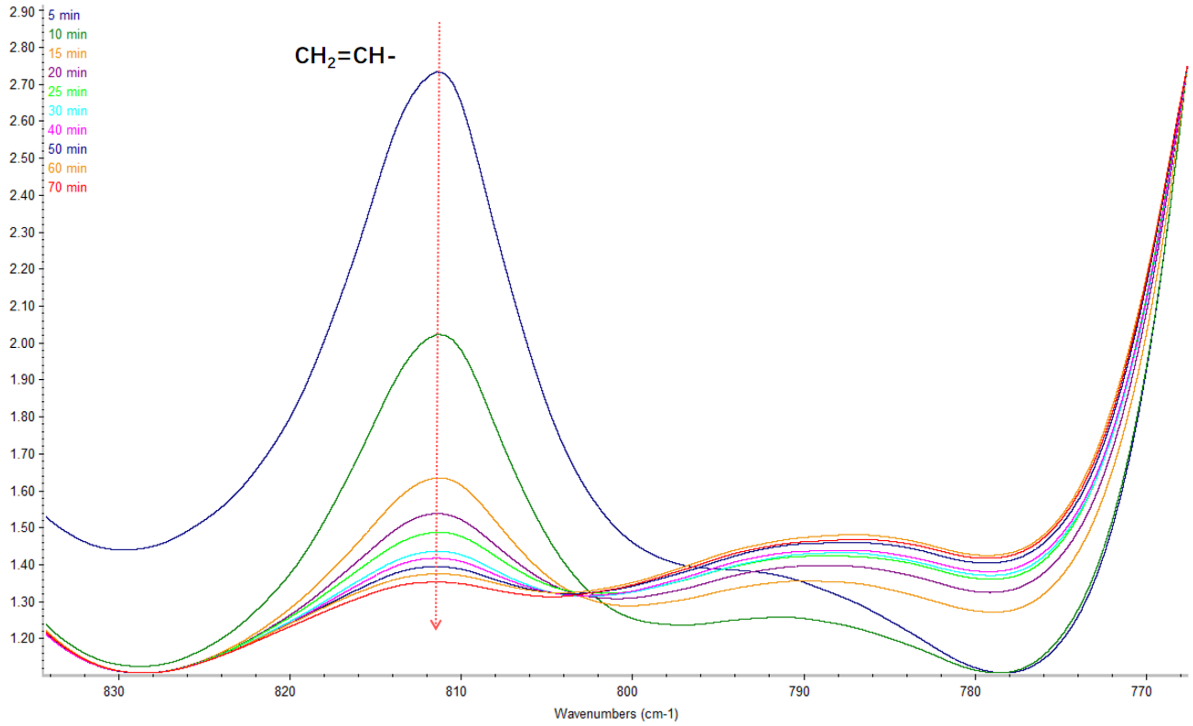

Figure S2. FTIR spectra monitoring the amine consumption during the reaction

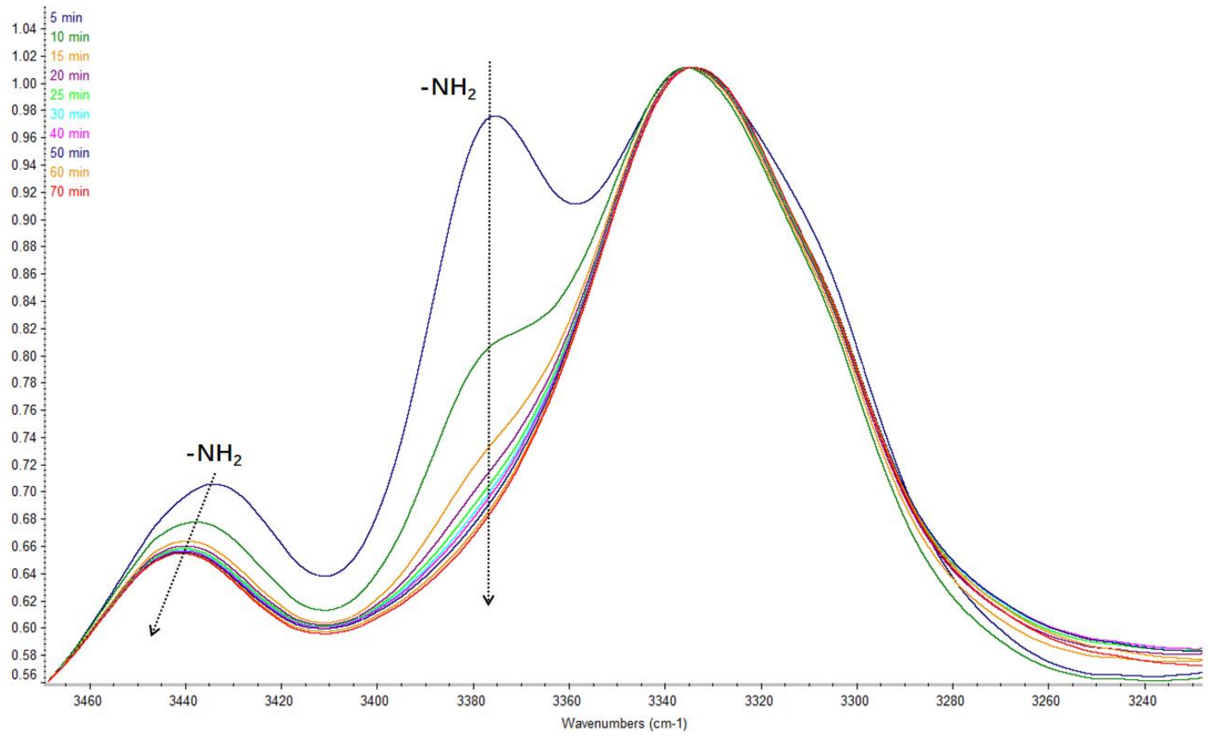

Figure S3. Mechanical stretchability of partially-cured gel, highlighting an exceptional durability up to the tensile strain of 1000%

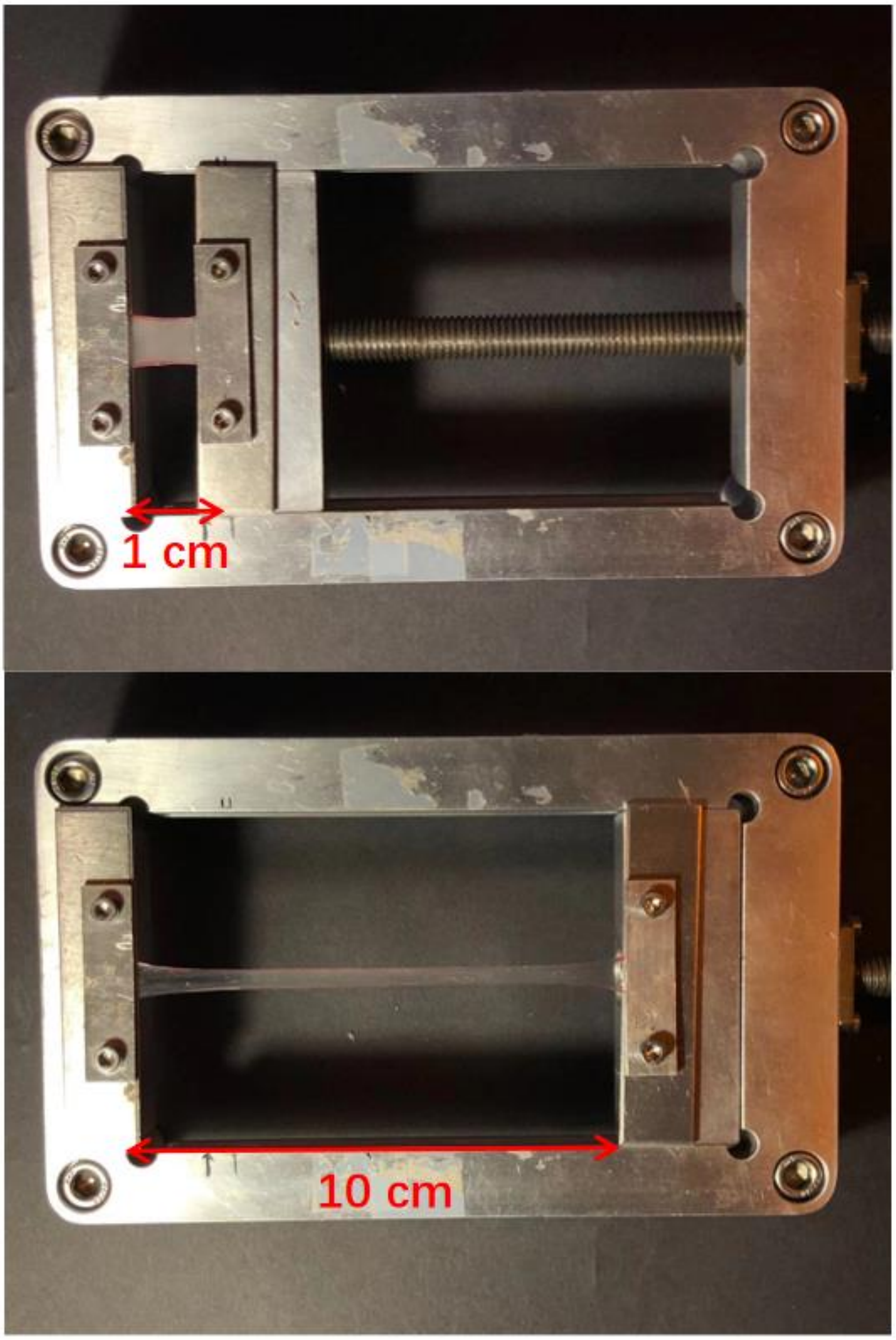

Figure S4. FTIR spectral analysis of free and bonded NH [1]

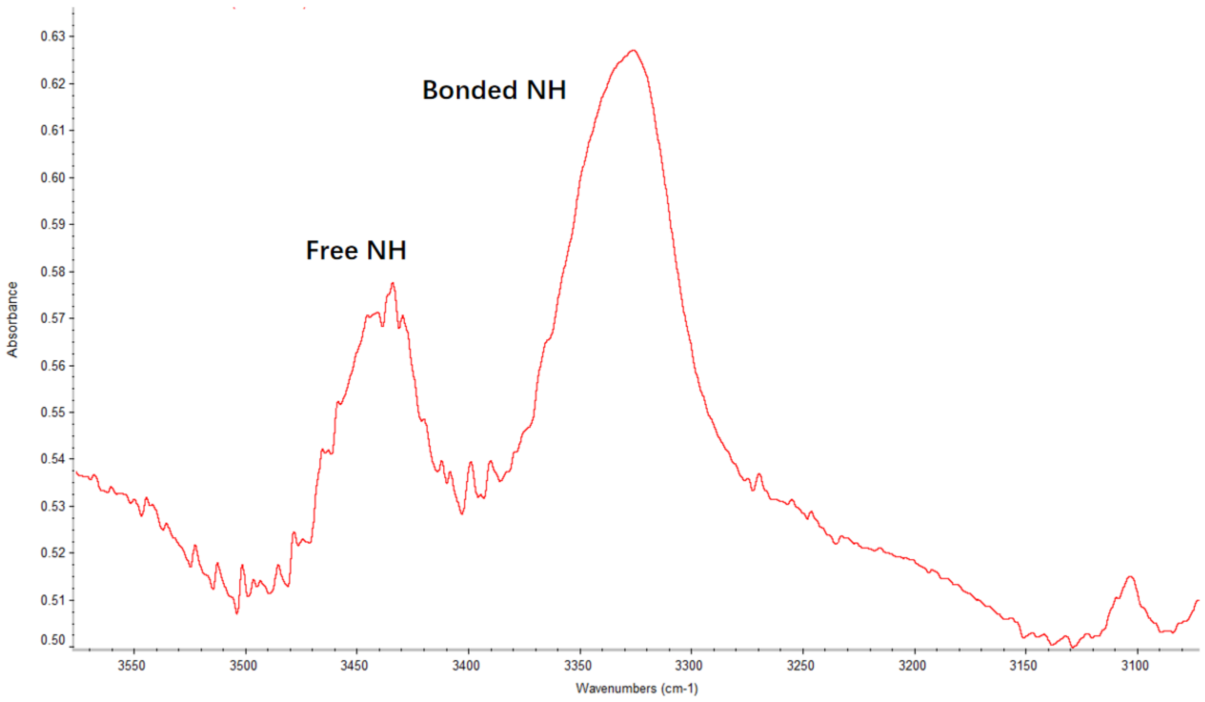

Figure S5. Variation of nematic order parameter Q during cooling from isotropic phase at a rate of cooling 10°/min. We used the theoretical equation relating the spontaneous load-free elongation of monodomain sample (strain ε) to the underlying order Q: $(1+\varepsilon)^3 = 1 + C \cdot Q$, which was extensively tested [2], and for which we had an independent X-ray measurement at room temperature fixing the constant *C*=5.6.

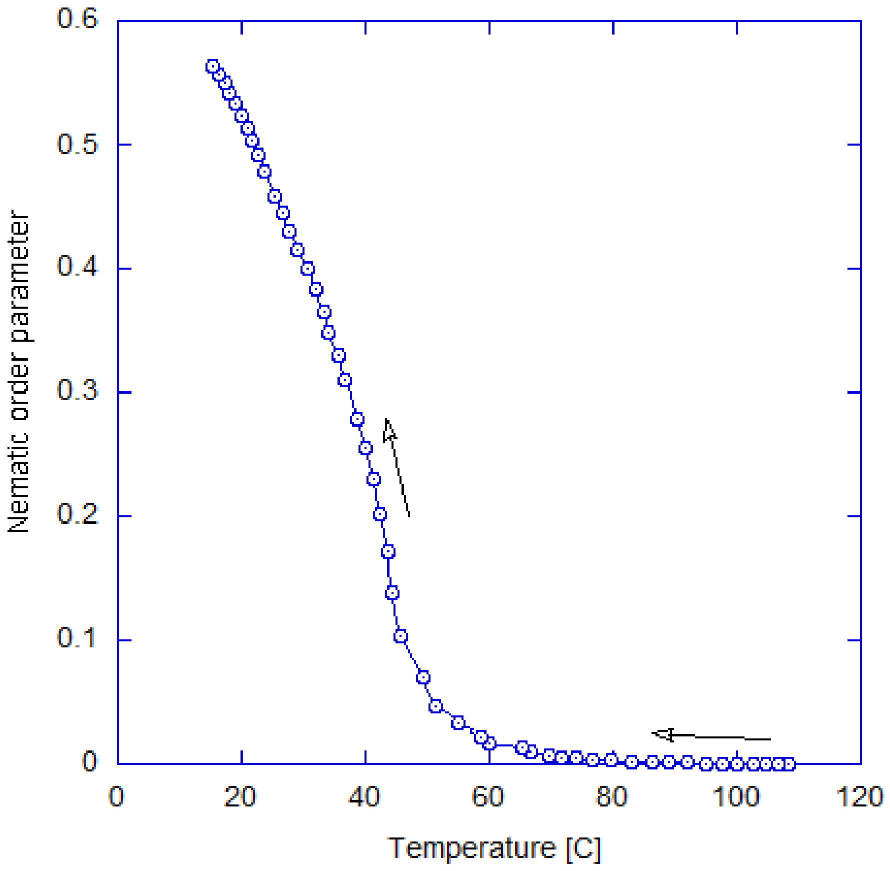

References:

[1] F. C. Wang, M. Feve, T. M. Lam, J. P. Pascault, *Journal of Polymer Science Part B: Polymer Physics* **1994**, 32, 1305.

[2] S. M. Clarke, A. Hotta, A. R. Tajbakhsh, E. M. Terentjev, Physical Review E 2001, 64, 061702.
